# Supplementary material for: Neofunctionalization of Glycolytic Enzymes: An Evolutionary Route to Plant Parasitism in the Oomycete Phytophthora nicotianae
Source: Microorganisms. 2022 Jan 25;10(2):281. doi: 10.3390/microorganisms10020281 (PMC8879444; doi:10.3390/microorganisms10020281)
Supplement: Supplementary file 1 [file microorganisms-10-00281-s001.zip › Kuhn-Supplementary Figures.pdf]

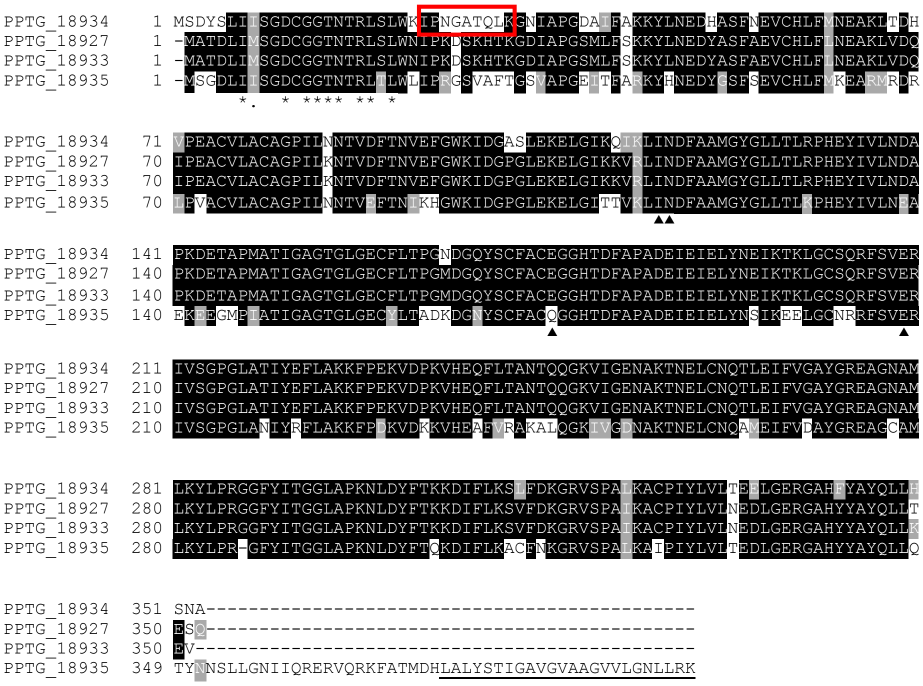


**A**

| PPTG_18927 | | 99.7 | 98 | 90.8 |
|---|---|---|---|---|
| PPTG_18933 | 99.7 | | 90.8 | 90.8 |
| PPTG_18934 | 90.7 | 90.7 | | 91.6 |
| PPTG_18935 | 77 | 76.8 | 76.9 | |
| | PPTG_18927 | PPTG_18933 | PPTG_18934 | PPTG_18935 |

**B**

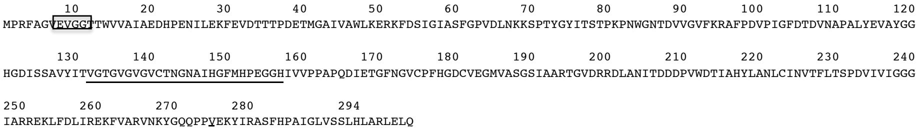


**C**

**Supplementary Figure S1**. Characteristics of *P. nicotianae* hexose kinases. (A) Alignment of predicted glucokinases. Identical residues are boxed in black, whereas conservative changes are shown in grey. Sequences were aligned using MUSCLE implemented in MEGA. The PTS2 recognition motif of PPTG_18934 (GKI) is indicated (red box) and the transmembrane domain of PPTG_18935 (GKIII) GKIII is underlined. Residues important for ATP and glucose binding (Lunin et al., 2004) are indicated by asterisks and black triangles, respectively. (B) Amino acid identity between the deduced proteins was calculated following alignment of the entire sequences. Values under the diagonal represent percent identity and values above the diagonal correspond to percent similarity. (C) Fructokinase. The signature domain (IPR000600) is underlined and the N-terminal ATP-binding motif (EXGXT) is boxed.

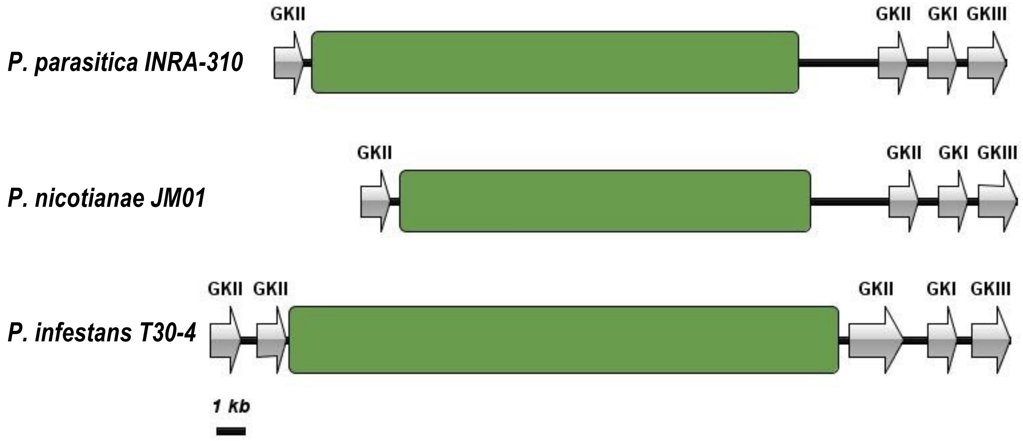


**Supplementary Figure S2.** Schematic representation of the GK gene cluster in *P. nicotianae (*INRA-310 and JM01*)* and *P. infestans*. The region containing footprints of transposable elements is represented by a green box.

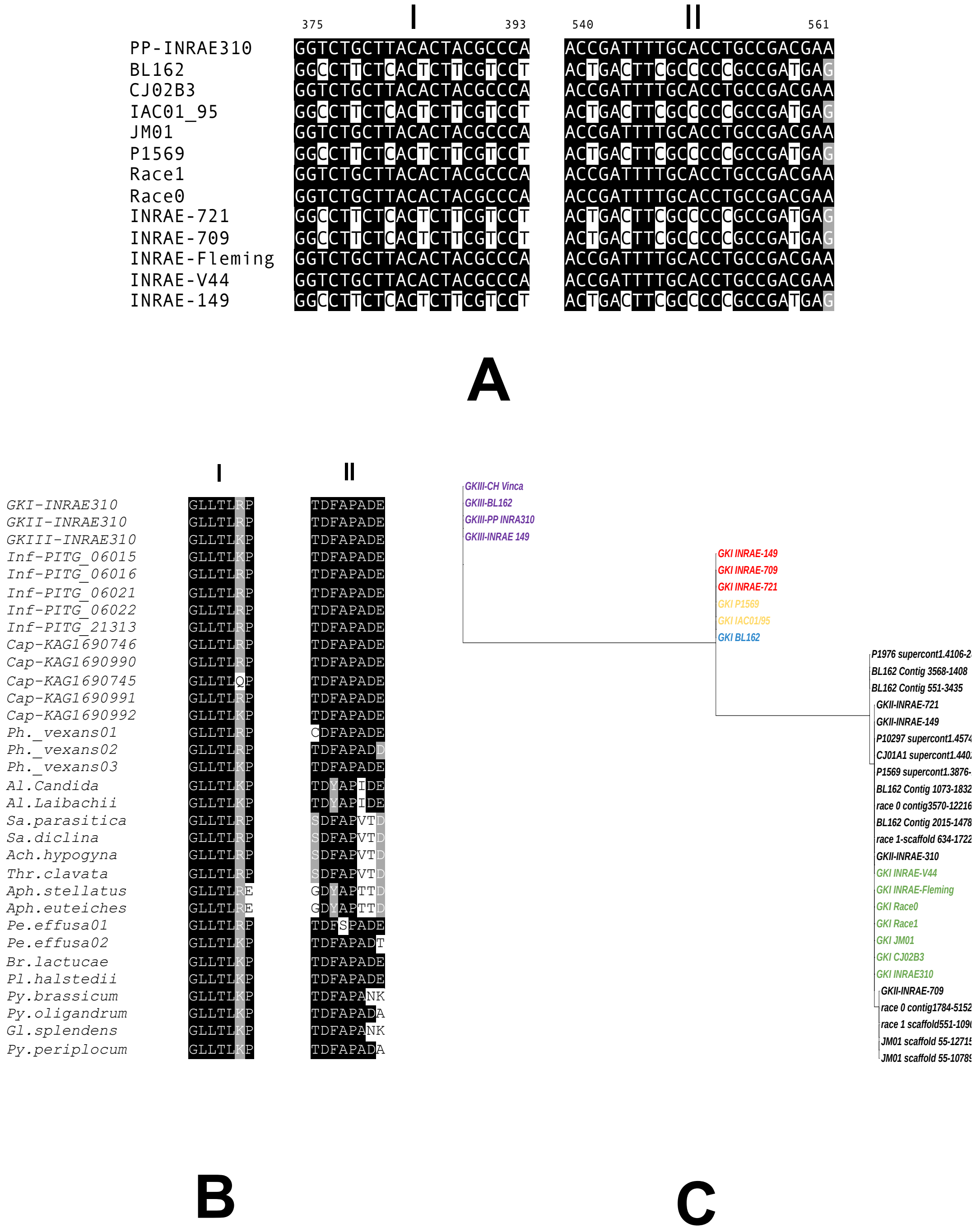


**Supplementary Figure S3. Diversification of GKI sequences.** A: local alignment of nucleotide sequences retrieved from GKI genes of *P. nicotianae* isolates from various origins (see Supplementary Table S1). Numbering refers to the entire length of the ORFs. B: Alignment of the translated boxes of Figure S3A on a range of oomycete GK sequences, including *P. nicotianae*, *P. infestans* (Inf), and *P. capsici* (Cap). Other abbreviations are depicted in Figure 2. C: Phylogenetic reconstruction of concatenated boxI + boxII nucleotide sequences from *P. nicotianae*. GKI-derived sequences are presented according to their host of origin, which are tobacco (green), tomato (red), citrus (yellow) and ornamentals (brown). GKII sequences are represented in black and GKIII sequences are represented in blue.

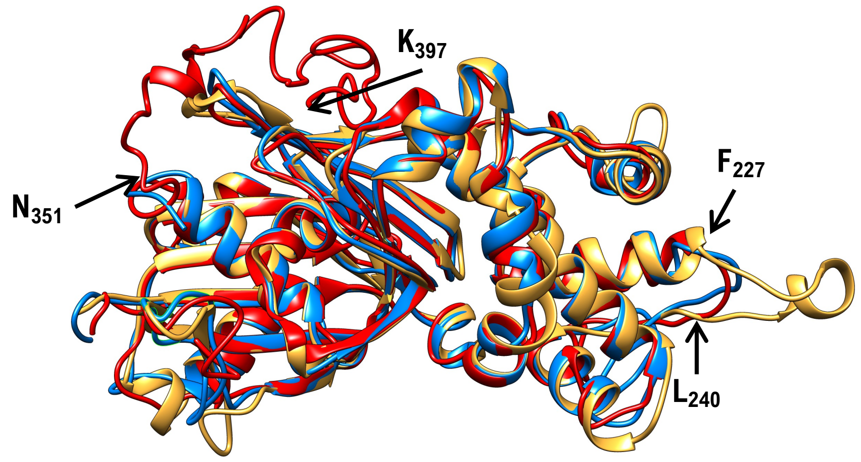


**Supplementary Figure S4**. Superposed 3D-structure models of GKI (blue), GKII (yellow) and GKIII (red). Structures were modelled as described in Figure 1. Structures were superposed using POSA and rendered using ChimeraX.

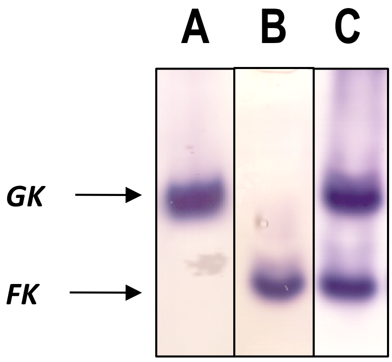


**Supplementary Figure S5**. Separation of glucokinase and fructokinase activities from *P. nicotianae* by electrophoresis in acrylamide gels under non-denaturing conditions. Proteins (50 μg) were loaded on the gel and enzymatic activity was revealed as indicated in Supplementary Materials using 10 mM glucose (A), 10 mM fructose (B) or glucose and fructose at a final concentration of 10 mM each (C).

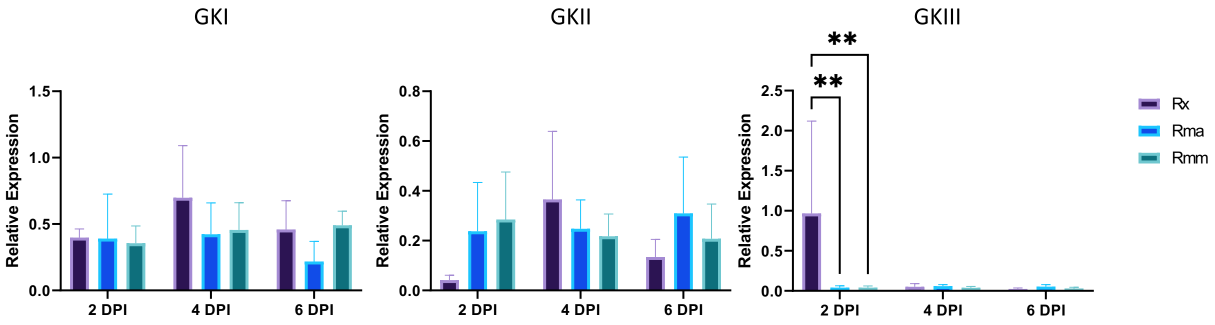


**Supplementary Figure S6**. Kinetics of GK transcripts accumulation during root invasion. Tomato (2 varieties) and tobacco (one variety) were inoculated with zoospores from 3 and 4 strains, respectively. Samples were collected at 2, 4 and 6 dpi and expression of GK genes was evaluated relative to the expression of the constitutive UBC gene. (A). Values were pooled according to plant variety. Statistical analyses were performed using a 2way ANOVA test. Rx: xanthii (tobacco); Rma: Marmande (tomato); Rmm: moneymaker (tomato).
