# Supplementary material for: Neofunctionalization of Glycolytic Enzymes: An Evolutionary Route to Plant Parasitism in the Oomycete Phytophthora nicotianae
Source: Microorganisms. 2022 Jan 25;10(2):281. doi: 10.3390/microorganisms10020281 (PMC8879444; doi:10.3390/microorganisms10020281)
Supplement: Supplementary file 1 [file microorganisms-10-00281-s001.zip › Kuhn-Supplementary materials.pdf]

Supplementary File

Neofunctionalization of glycolytic enzymes is an evolutionary route to parasitism in the oomycete *Phytophthora nicotianae*

Marie-Line Kuhn [1#], Jo-Yanne Le Berre [1#], Naima Minet [1] and Franck Panabières [1,*]

This Supplementary File includes:

Supplementary Materials and Methods

Supplementary Materials and Methods

Detection of hexose kinase activities

Four-day mycelia cultivated on liquid V8 medium were harvested, rinsed with sterile water and frozen in liquid nitrogen. Powdered mycelium was suspended in two volumes of 0.1M Tris-HCl, pH 7.5, 5mM DTT, 10% glycerol, 0.1mM PMSF. After centrifugation for 15 min at 16,500 x g at 4°C, the supernatant, corresponding to the cytosolic fraction, was divided into aliquots and stored at -20°C. Protein concentration in the extracts was determined by the Bradford method (Bio-Rad) using bovine serum albumin as standard. A 50-µg sample of cytosolic proteins was loaded on non-denaturating, 10% acrylamide gels in 0.375M Tris-HCl, pH 8.8. Ammonium persulfate was replaced by riboflavin to allow gel polymerization. For detection of glucokinase activity, glucose was added in the gel at a 50 mM final concentration as recommended [2]. After electrophoresis, hexose kinase activities were determined by coupling the phosphorylation of either glucose or fructose to the reduction of NAD by glucose-6-phosphate dehydrogenase (G6PDH) [3]. The staining mixture contained 5 mM ATP, 8 mM $MgCl_2$, 2 mM ß-NAD, 10 mM glucose or fructose in a 0.1M $NaPO_4$ buffer (pH 7.5). The enzymatic reaction was initiated by addition of a mix containing 1.5 u/ml *Leuconostoc mesenteroides* G6PDH (Sigma), 0.2 mM PMS (phenazine methosulfate, Sigma), and 0.6 mM MTT (3-[4,5-dimethylthiazol-2yl]-2,5-diphenyltetrazolium bromide, Sigma). When using fructose as a substrate, 1u/ml phosphoglucose isomerase (PGI) was added. For the evaluation of mannokinase activity, the reaction mixture prepared for fructokinase was supplemented with 1 unit/ml phosphomannose isomerase (PMI) and mannose to a final concentration of 2mM. For proper detection of enzymatic activities in the gels, the mixture described above was supplemented with a low melting agarose solution (to a final concentration of 0.66%) maintained at 42°C and overlaid on the acrylamide gels that were incubated at 24°C in the dark.

Expression and purification of recombinant hexose kinases in E. coli and kinetic analyses

The coding regions of *PpGK1*, *PpGK2*, *PpGK3* and *PpFK1* were inserted into the *E. coli* expression vector pET28 (Invitrogen), and cloned in *E. coli* Rosetta DE3 strain (Novagen). Bacterial cultures were conducted at 37°C to an in LB medium containing kanamycin (30 µg/ml) and chloramphenicol (34 µg/ml). Expression of the recombinant polypeptides was induced at an A_{600} of 0.7 by the addition of 1 mM isopropylthio-B-galactosidase (IPTG) and the growth was continued for 3h at 37°C. Cells were harvested by centrifugation at 4,000xg for 10 min at 4°C and the pellets were resuspended in buffer A (20mM Na_2HPO_4, 0.5M NaCl, ph 7.4, 25 mM imidazole) supplemented with 1mM PMSF. Lysozyme was added to a final concentration of 0.2 mg/ml. After a 30-min incubation at room temperature with gentle shaking, the preparation was centrifuged for 20 min at 16.000xg at 4°C. The resulting preparation was applied to a HisTrap FF crude column (GE Healthcare) equilibrated with buffer A. According to the manufacturer's recommendation, the column was washed with buffer A, and elution was achieved with the same buffer plus 0.5 M imidazole. Glucokinase and fructokinase activities were assessed on the crude extract before purification on the column and after elution as described above. The active fractions were dialyzed against 0.1 M Na_2HPO_4, pH 7.5 at 4°C and stored at -20°C. Purity of recombinant enzymes was checked by SDS-PAGE in 10% acrylamide gels followed by Comassie blue staining.

The K_m values for hexoses and ATP were calculated using Prism (GraphPad, San Diego, CA) software by non-linear regression analysis. The 1-ml assay mixtures contained 2 mM $MgCl_2$, 2 mM NAD, 0.1 M Na_2HPO_4, pH 8.0, 1 unit G6PDH, 400 ng recombinant protein, and varying concentrations of glucose and ATP. Glucose concentrations varied from 0.02 mM to 2 mM in the presence of 2 mM ATP. To assess the sugar substrate specificity of the recombinant glucokinases, glucose was replaced by fructose whose concentrations ranged from 0.05 mM to 5 mM. The K_m for ATP was determined with increasing ATP concentrations in a 0.05 mM- 1 mM range, in the presence of 0.55 mM, 0.35 mM glucose for recombinant PpGK1 and PpGK2, respectively (see results). The reaction was initiated by adding glucose and monitored spectrophotometrically at 340 nm. For PpGK3, Na_2HPO_4 was replaced by Tris-HCl pH 8.0, after initial, comparative assays. For the recombinant PpFK1, the K_m for fructose was determined with

concentrations varying from 0. 075 mM to 0.4 mM in an assay mixture containing 2 mM $MgCl_2$, 2 mM ATP, 2 mM NAD, 1 unit PGI and 1 unit G6PDH. To calculate the K_m values for mannose, the mixture developed for PpFK1 was complemented with 1 u PMI and a 2-60 mM mannose range. The nucleotide specificity was determined via the standard enzyme assay and replacing ATP by various nucleotides at similar concentrations (see results). Possible inhibition by glucose-6-phosphate (G6P) was evaluated by monitoring the NADH oxidation at 340 nm coupled to the L-lactate dehydrogenase (LDH) reaction via pyruvate kinase (PK). We slightly deviated from the published procedure [3]. Briefly, reaction was done in 0.1 M Tris-HCl or Na_2HPO_4, pH 7.5 (depending on the tested enzyme, see results), 10 mM MgCl2, 40 mM KCl, 2 mM DTT, 0.2 mM NADH, 5 mM phosphoenolpyruvate (PEP), 2 mM ATP, 1 mM glucose, 1-10 mM G6P, 5 unit LDH and 5 unit PK.
